# Refractory pediatric actinic prurigo successfully treated with upadacitinib: A case report

**DOI:** 10.1177/2050313X251341558

**Published:** 2025-05-13

**Authors:** Florence Morissette, Jérome Coulombe

**Affiliations:** 1 Dermatology Division, Department of Medicine, CHUM, University of Montreal, QC, Canada; 2 Dermatology Division, Department of Pediatrics, CHU Sainte-Justine, Montreal, QC, Canada

**Keywords:** pediatrics, photodermatitis, treatments

## Abstract

Actinic prurigo is a rare photodermatosis, usually occurring during childhood. Pruritic papulonodules on sun-exposed areas are frequently associated with cheilitis and conjunctivitis. The most widely accepted pathophysiology to date is a type IV hypersensitivity reaction triggered by ultraviolet radiation. This case report describes a 12-year-old boy suffering from refractory actinic prurigo, who was successfully treated with the janus kinase inhibitor upadacitinib.

## Introduction

Actinic prurigo is a rare photodermatosis, usually occurring during childhood. Pruritic erythematous papules and nodules erupt in sun-exposed areas. The face and distal limbs are typically affected, although the involvement of covered sites has been described. Cheilitis and conjunctivitis are frequently associated. Actinic prurigo is triggered by ultraviolet (UV) exposure, eliciting an immunologic response in the skin.^1^ A strong association with the human leukocyte antigen (HLA) DR4 subtype DRB1*0407 has been reported.^2^ Photoprotection and topical corticosteroids are the first-line treatments, although they are rarely sufficient. Systemic therapies include corticosteroids, thalidomide, azathioprine and cyclosporine, with mixed responses.^1^ We report the case of a 12-year-old boy affected by refractory actinic prurigo since the age of 7, successfully treated with upadacitinib.

## Case report

**Figure 1.**
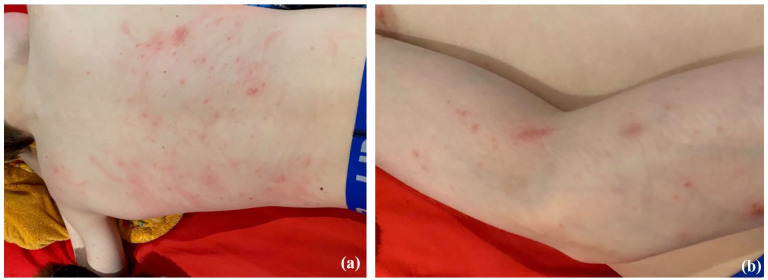
Actinic prurigo skin lesions. Erythematous and excoriated papules on back (a) and arm (b) in a 12-year-old boy affected by actinic prurigo after a shirtless sunny day.

The patient was known for herpes labialis, IgA and C2 deficiencies. He developed pruritic papules on his face, neck, trunk and extremities during summertime (see Figure 1). On examination, subtle cheilitis and conjunctivitis were noted. Systemic symptoms were absent. A complete workup to rule out photodermatoses revealed antinuclear antibody 1/320, elevated ds-DNA at 910 UI/mL and normal C3, C4, ENA and porphyrin levels. Multiple skin biopsies showed subacute spongiotic dermatitis with a negative lupus band test. Pediatric rheumatology was consulted, and a diagnosis of lupus was excluded. Genetic testing on blood confirmed a positive HLA-DRB1*0407 status compatible with actinic prurigo.

In subsequent summers, his skin photosensitivity was so intense that he would only go outside with full coverage, greatly impairing his quality of life. Broadband sunscreen applications provided only partial control and led to weariness. He failed multiple therapies, including topical potent corticosteroids, topical tacrolimus, polypodium leucotomos, beta-carotene, hydroxychloroquine, methotrexate, mycophenolate mofetil and dupilumab. At 12 years old, we attempted compassionate care with upadacitinib 15 mg daily, resulting in rapid improvement of its photosensitivity without side effects. Omitting a dose on a sunny day was associated with a relapse of skin lesions and pruritus, but resuming the medication led to rapid symptom relief within a few hours. Given the quick effect of upadacitinib, the patient decided to tailor upadacitinib intake as needed. He is now able to play outdoors for full days without relapse of bothersome symptoms.

## Discussion

The immunologic response elicited by UV exposure in actinic prurigo is not fully understood. A type IV hypersensitivity reaction, Th2-mediated and driven by interleukin-4 (IL-4), IL-5 and IL-13, promoting IgE production by B cells and activating eosinophils and mast cells, has been suggested. Eosinophilic and monocytic infiltrates in skin biopsies^3^ and elevated circulating IgE blood levels sometimes found in patients with moderate-to-severe actinic prurigo^4^ support this hypothesis. Upadacitinib, a reversible janus kinase (JAK) inhibitor targeting the JAK1 enzyme, is FDA-approved for use in individuals over 12 years old for many immunologic diseases, including moderate-to-severe atopic dermatitis. JAKs are non-receptor tyrosine protein kinases mostly associated with multiple cytokine receptors.^5^ Tyrosine kinases enable signal transduction by transferring a phosphate group from ATP to tyrosine residues of specific proteins, leading to the activation of various immune cascades, including the Th2 pathway.^5^ We hypothesize that upadacitinib is rapidly effective in actinic prurigo due to its capacity to reversibly block the Th2 pathway in addition to a wide range of other pro-inflammatory mediators. Two cases of patients suffering from actinic prurigo successfully treated with the JAK inhibitor baricitinib have been reported.^6,7^

In conclusion, upadacitinib demonstrated a complete and rapid relief of skin lesions and pruritus in our patient with refractory actinic prurigo, with intermittent intake tailored to symptom intensity. Compared to other systemic medications and biologics, upadacitinib’s short onset of action and half-life allow a flexible therapeutic regimen, potentially limiting side effects and decreasing the frequency of blood monitoring. Its oral administration is also advantageous for the pediatric population.
